# Test-Retest Reliability and Informant Consensus Pilot Study of the BRIEF-A in the Non-Clinical Spanish Population

**DOI:** 10.1192/j.eurpsy.2025.535

**Published:** 2025-08-26

**Authors:** I. González Herrera, R. Sandoval Rodríguez, A. B. Calvo Calvo, J. M. Espejo-Saavedra Roca

**Affiliations:** 1 Personality, Assessment and Clinical Psychology Department; 2 Universidad Complutense de Madrid; 3 Hospital Universitario 12 de Octubre, Madrid, Spain

## Abstract

**Introduction:**

Executive functions (EEFF) are different cognitive aspects that allow us to find solutions and adapt to changes. There are several traditional instruments that assess these processes, but they are difficult to generalize to the subject’s real environment.

**Objectives:**

To analyze the test-retest reliability of the adaptation of the BRIEF-A to the Spanish population with a non-clinical sample, as well as studying the informant consensus between the Self-report and Informant report forms that this instrument presents.

**Methods:**

The questionnaire has been administered to 58 subjects from the general population (Self-report version) and 58 informants who adequately knew each subject (Informant report version) at baseline and at 4 weeks follow-up in order to study the test-retest reliability. Statistical analysis was carried out using the Pearson Correlation Coefficient to study the test-retest reliability. Self-reports and Informant reports mean scores were also compared using the Student’s T-Test for paired samples.

**Results:**

The calculated correlations were significant for all the scales and indices in both the Self-report and the Informant report versions, and relatively strong in all the cases with values ranging from 0.62 to 0.89 except for the Task Monitor scale in the Self-report form, which presented a moderate correlation (*r* = 0.31). When it comes to the T-Test, mean differences between the two samples were also statistically significant in all the cases.

**Conclusions:**

Test-retest reliability of the BRIEF-A is adequate in the non-clinical Spanish population, and the scores obtained in the first occasion remain relatively stable in the second. Furthermore, the informant consensus is observed to be low, therefore showing the utility of gathering data from different informants, since that can provide a better approach of subjects EEFF. Further research with clinical population would be necessary to validate this tool and perform a complete assessment.

**Disclosure of Interest:**

None Declared

